# PU-91 drug rescues human age-related macular degeneration RPE cells; implications for AMD therapeutics

**DOI:** 10.18632/aging.102179

**Published:** 2019-09-02

**Authors:** Sonali Nashine, Sudhakar R. Subramaniam, Marilyn Chwa, Anthony Nesburn, Baruch D. Kuppermann, Howard Federoff, M. Cristina Kenney

**Affiliations:** 1 Department of Ophthalmology, Gavin Herbert Eye Institute, University of California Irvine, Irvine, CA 92697, USA; 2 Department of Neurology, University of California Irvine, Irvine, CA 92697, USA; 3 Cedars-Sinai Medical Center, Los Angeles, CA 90048, USA; 4 Department of Pathology and Laboratory Medicine, University of California Irvine, Irvine, CA 92697, USA

**Keywords:** age-related macular degeneration (AMD), RPE, PGC-1α, mitochondria, FDA-approved drugs

## Abstract

Since mitochondrial dysfunction is implicated in the pathogenesis of AMD, this study is based on the premise that repurposing of mitochondria-stabilizing FDA-approved drugs such as PU-91, might rescue AMD RPE cells from AMD mitochondria-induced damage. The PU-91 drug upregulates *PGC-1α* which is a critical regulator of mitochondrial biogenesis. Herein, we tested the therapeutic potential of PU-91 drug and examined the additive effects of treatment with PU-91 and esterase inhibitors i.e., EI-12 and EI-78, using the *in vitro* transmitochondrial AMD cell model. This model was created by fusing platelets obtained from AMD patients with *Rho^0^* i.e., mitochondria-deficient, ARPE-19 cell lines. The resulting AMD RPE cell lines have identical nuclei but differ in their mitochondrial DNA content, which is derived from individual AMD patients. Briefly, we report significant improvement in cell survival, mitochondrial health, and antioxidant potential in PU-91-treated AMD RPE cells compared to their untreated counterparts. In conclusion, this study identifies PU 91 as a therapeutic candidate drug for AMD and repurposing of PU-91 will be a smoother transition from lab bench to clinic since the pharmacological profiles of PU-91 have been examined already.

## Introduction

The incidence of Age-related Macular Degeneration (AMD) is increasing at an alarming rate in elderly population in the United States. Per the National Eye Institute projection, the estimated number of AMD patients is expected to rise to 5.44 million by 2050. Most AMD cases occur among Caucasian Americans, followed by Hispanic and other populations. AMD – a disease which damages the macula and affects central vision, remains a leading cause of uncorrectable vision loss in the United States [1]. Despite intensive study, a limited number of FDA-approved treatment options are available for treatment of AMD. Anti-VEGF drugs such as Ranibizumab, Bevacizumab, and Aflibercept have been demonstrated to reduce choroidal neovascularization in AMD. Therefore, injections of these anti-VEGF drugs into the vitreous cavity are by far the most viable treatment option available for wet AMD [2]. Furthermore, AREDS (Age-Related Eye Disease Study) supplements, which are over-the-counter antioxidant/zinc supplements, are known to slow down the progression of AMD [3].

Mitochondrial (mt) DNA damage due to mutations or oxidative stress has long been implicated in the development of AMD [4]. Mitochondrial DNA damage induces ARPE-19 cells to secrete pro-inflammatory cytokines associated with onset and progression of AMD [5]. Macular RPE cells from aged and AMD human donor eyes have higher frequencies of mtDNA lesions and mtDNA genomic heteroplasmic mutations, compared to their age-matched controls. AMD severity has been associated with decreased expression of a DNA repair enzyme OGG1, which is involved in excision repair of oxidatively-damaged DNA. Accumulation of mtDNA lesions and reduced DNA repair capacity contribute to loss of RPE cells in AMD and aging retinas [6]. Therefore, several mitochondria-targeting therapeutic molecules have been identified in the hope of rescuing mtDNA and subsequently RPE cells in AMD. For instance, a mitochondria-targeted antioxidant SkQ1 prevents AMD progression in an *in vivo* model of AMD [7]. A mitochondria-targeting peptide called MTP-131 (Bendavia) targets cardiolipin and improves mitochondrial function [8]. Furthermore, our recent work has shown that Humanin G (HNG) which is a more potent variant of Humanin, a mitochondrial-derived peptide, rescues AMD RPE cybrid cells *in vitro* [9]. In that study, we demonstrated that mitochondria from AMD patients were dysfunctional compared to the normal mitochondria which were derived from age-matched normal subjects. Mitochondrial DNA damage was evidenced by significant reduction in mtDNA copy numbers and higher numbers of mtDNA lesions in the AMD cybrids compared to that in the normal cybrids. Furthermore, decreased expression of mitochondrial transcription and replication genes suggesting impaired mitochondrial transcription and replication was observed in the AMD cybrid cells compared to their normal counterparts. Moreover, this work with AMD cybrids revealed higher mitochondrial superoxide generation and reduced mtGFP fluorescent staining in AMD cybrids compared to normal cybrids [9]. Therefore, our previous findings established substantive mitochondrial damage in AMD cybrid cell lines compared to the normal cybrid cell lines which served as controls.

Since mitochondrial biogenesis is influenced by PGC-1α (Peroxisome-proliferator-activated receptor γ Coactivator-1α) expression and activity [10,11], numerous pharmacological interventions in retinal and neurodegenerative diseases have been directed toward PGC-1α upregulation [12–15].

The purpose of this study was to test the following hypothesis: PU-91, an FDA-approved mitochondrion-stabilizing drug, will protect RPE cells in an *in vitro* macular degeneration model. PU-91 is a pro-drug that when metabolized is PPARα ligand and which was developed for the treatment of dyslipidemia. The drug is estimated to have seen >5 million-years of patient exposure and remains an effective agent for certain dyslipidemias. PU-91, not its metabolite, is the chemical matter that produces the upregulation of PGC-1α (data not shown, manuscript in preparation). Our *in vitro* AMD model was created by fusion of mitochondria-deficient APRE-19 (*Rho^0^*) cell line with platelets isolated from AMD patients. The resulting AMD RPE transmitochondrial cybrids have identical nuclei derived from ARPE-19 cells but mitochondria from different AMD patients (Supplementary Table 1). We investigated the effect of PU-91 drug on mitochondrial biogenesis, apoptosis, oxidative stress, mitochondrial membrane potential, mitochondrial superoxide production, mtGFP staining, and finally examined the additive effects of PU-91 and esterase inhibitors i.e., EI-12 and EI-78 in AMD RPE transmitochondrial cybrids. Our findings demonstrated that PU-91 preserved AMD mitochondrial function and integrity, and protected AMD RPE cybrids against oxidative stress-induced and mtDNA-induced apoptotic cell death. This study suggests a potential role for PU-91 as a candidate drug for AMD treatment. Since PU-91 is an FDA-approved drug, its repositioning for treatment for AMD would encounter a smoother pathway because its side effects and other risks are already known. This shortens considerably the journey from lab bench to clinic. However, further studies are required to establish the merit of PU-91 as a cytoprotective molecule in AMD.

## RESULTS

### PU-91 increases mitochondrial DNA copy number and upregulates the genes involved in mitochondrial biogenesis pathway in AMD RPE cells

Mitochondrial biogenesis involves the orchestration of expression of multiple nuclear encoded genes, in large part, mediated through the transcriptional action of *PGC-1α* gene product in concert with others. As PU-91 is posited to upregulate mitochondrial biogenesis, we sought to measure mitochondrial DNA (mtDNA) copy number and transcriptional outputs in AMD RPE cybrid cells treated with this repositioned drug.

**Figure 1:**
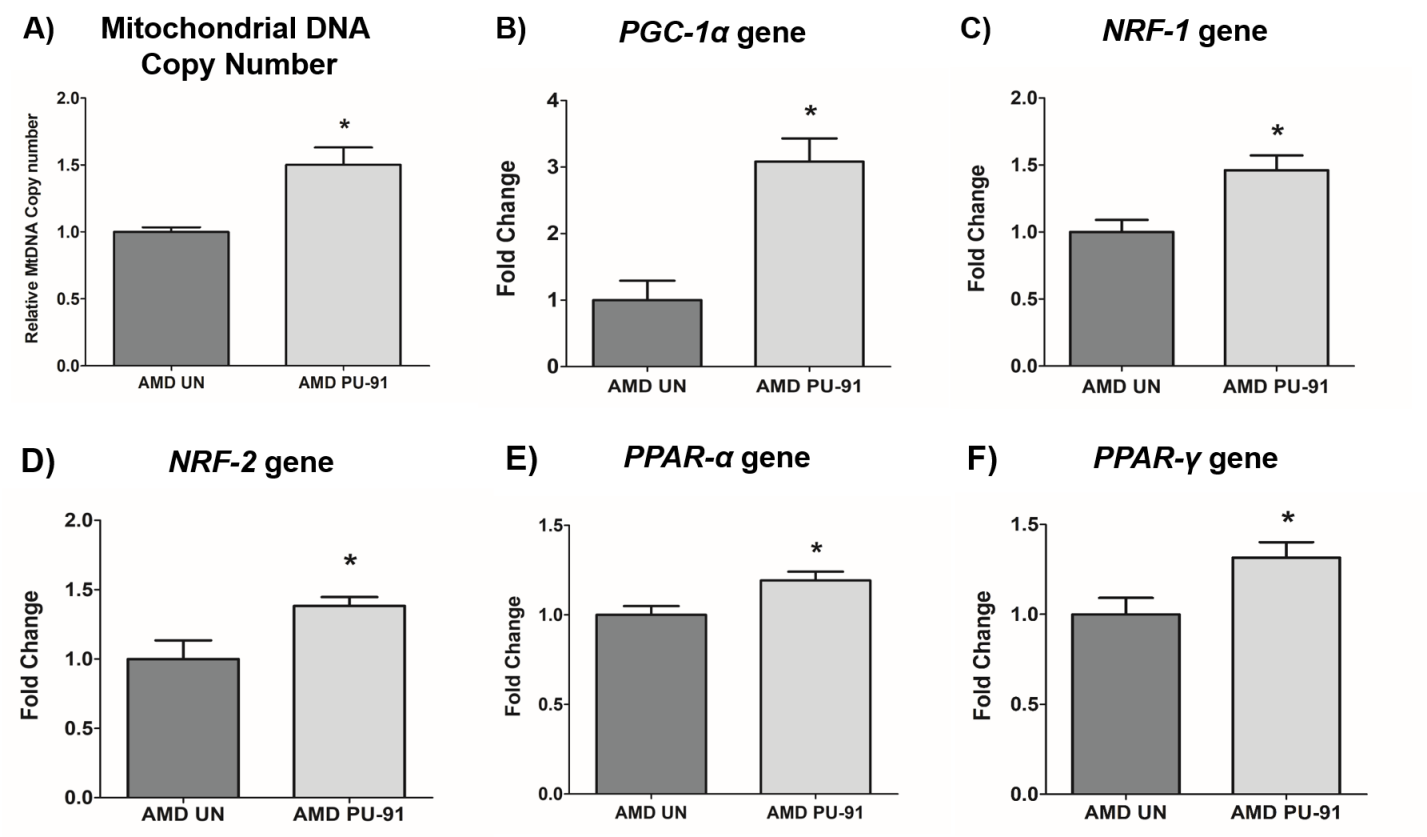
**PU-91 regulates the mitochondrial biogenesis pathway.** We used quantitative qRT-PCR to measure the relative mtDNA copy number (**A**), and the gene expression of markers of the mitochondrial biogenesis pathway such as *PGC-1α* (**B**), *NRF-1* (**C**), *NRF-2* (**D**), *PPAR-α* (**E**), and *PPAR-γ* (**F**). PU-91-treated AMD cybrids (AMD PU-91) had higher mtDNA copy numbers and increased gene expression levels of all the above-mentioned markers (p≤0.05, n=4-5). Data are presented as mean ± SEM and normalized to untreated (UN) AMD cybrids which were assigned a value of 1. Mann-Whitney test was used to measure statistical differences; *p≤0.05.

Accordingly, PU-91 significantly increased relative mtDNA copy numbers by 50% (*p*=0.03; AMD UN: 1 ± 0.03, n=4; AMD PU-91: 1.50 ± 0.13, n=4) (Figure 1A) and upregulated the gene expression of *PGC-1α* by 208% (*p=* 0.016; AMD UN: 1 ± 0.29, n=5; AMD PU-91: 3.08 ± 0.35, n=5) (Figure 1B), *NRF-1* by 46% (p= 0.03; AMD UN: 1 ± 0.09, n=4; AMD PU-91: 1.46 ± 0.1, n=4) (Figure 1C), *NRF-2* by 38% (p= 0.03; AMD UN: 1 ± 0.13, n=5; AMD PU-91: 1.38 ± 0.06, n=5) (Figure 1D), *PPAR-α* by 19% (p= 0.03; AMD UN: 1 ± 0.05, n=5; AMD PU-91: 1.19 ± 0.05, n=5) (Figure 1E), and *PPAR-γ* by 32% (p= 0.03; AMD UN: 1 ± 0.09, n=5; AMD PU-91: 1.32 ± 0.08, n=5) (Figure 1F) in AMD cybrids compared to their untreated counterparts.

### PU-91 improves mitochondrial function in AMD RPE cells

**Figure 2:**
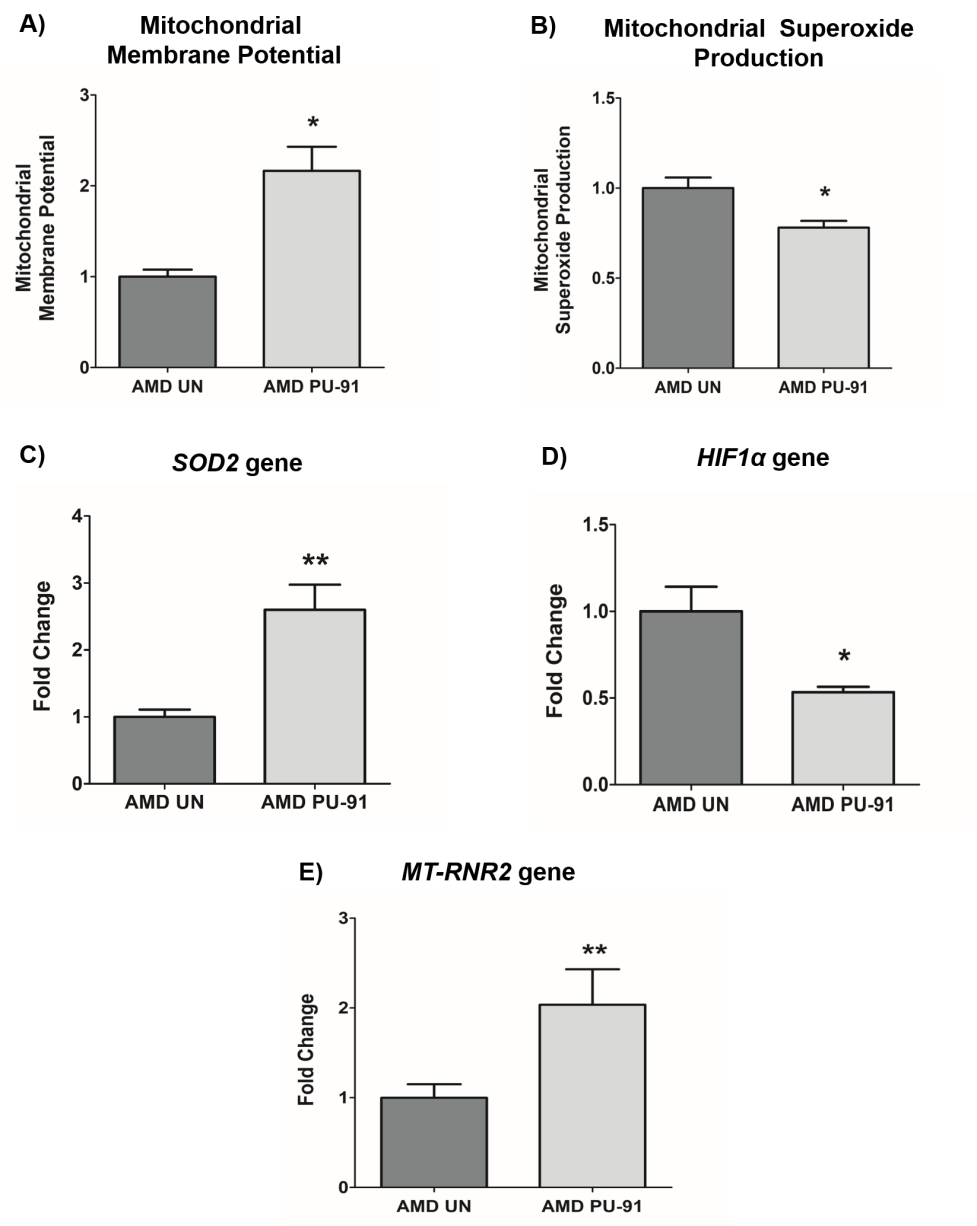
**PU-91 regulates mitochondrial function.** We used the fluorometric JC-1 assay and MitoSOX assay to measure mitochondrial membrane potential and mitochondrial superoxide production, respectively. Treatment with PU-91 led to elevated mitochondrial membrane potential (p≤0.05, n=3) (**A**) and reduced mitochondrial superoxide production (p≤0.05, n=3) (**B**) in AMD cybrids (AMD PU-91) compared to the untreated group (AMD UN). Furthermore, PU-91-treated AMD cybrids showed upregulation of the mitochondrial superoxide dismutase, *SOD2* gene (p≤0.05, n=5) (**C**) and reduced expression of *HIF1α* gene (p≤0.05, n=3-4) (**D**). (**E**) PU-91 upregulates *MT-RNR2* gene. Using TaqMan probe for the *MT-RNR2* gene, qRT-PCR analysis revealed that PU-91 increases *MT-RNR2* gene expression by 104% compared to untreated control (p≤0.05, n=5). Data are presented as mean ± SEM and normalized to untreated (UN) AMD cybrids which were assigned a value of 1. Mann-Whitney test was used to measure statistical differences; *p≤0.05, **p≤0.01.

It would be anticipated that transcriptional activation of genes that promote mitochondrial biogenesis would be accompanied by evidence of improved mitochondrial function. As shown in Figure 2, PU-91-treated AMD cybrid cells had increased mitochondrial membrane potential (JC-1 assay) (116% increase; *p*= 0.03; AMD UN: 1 ± 0.09, n=3; AMD PU-91: 2.16 ± 0.26, n=4), (Figure 2A), and significantly lower levels of mitochondrial superoxides (MitoSOX assay) (22% increase; *p*=0.04; AMD UN: 1 ± 0.06, n=4; AMD PU-91: 0.78 ± 0.038, n=4), (Figure. 2B). Furthermore, PU-91-treated AMD cells showed upregulation of *SOD2*, a mitochondrial antioxidant gene, by 160%, (p=0.0079; AMD UN: 1 ± 0.11, n=5; AMD PU-91: 2.6 ± 0.37, n=5) (Figure 2C), and reduced gene expression of *HIF1α* (47% increase; *p*=0.03; AMD UN: 1 ± 0.14, n=4; AMD PU-91: 0.53 ± 0.03, n=4) (Figure 2D). PU-91 upregulated *MT-RNR2* (Mitochondrially Encoded 16S rRNA) gene in AMD RPE cybrid cells. Treatment with PU-91 drug caused a 104% higher expression of *MT-RNR2* gene in AMD RPE cybrid cells (p=0.0079; AMD UN: 1 ± 0.15, n=5; AMD PU-91: 2.04 ± 0.39 n=5) (Figure 2E), suggesting that increased production of Mitochondrial Derived Peptides (MDPs) could be one of the mechanisms by which PU-91 rescues cells.

### PU-91 enhances mitochondrial GFP (mtGFP) fluorescence in AMD RPE cells

**Figure 3:**
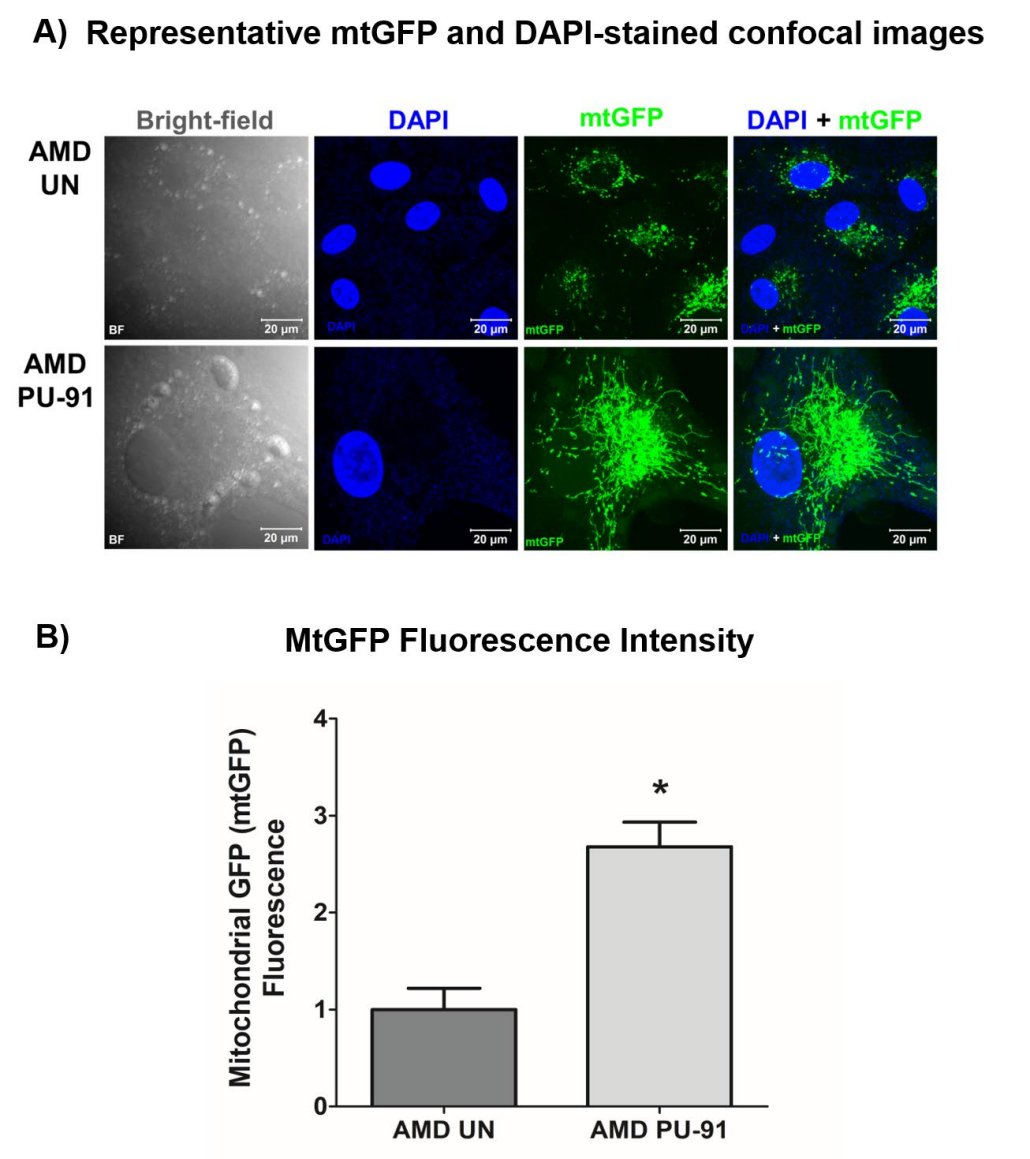
**PU-91 alters mitochondrial GFP fluorescence intensity.** Untreated (AMD UN) and PU-91-treated AMD cybrids (AMD PU-91) were stained with CellLight mitochondrial GFP stain followed by confocal imaging of cells. (**A**) Shows representative bright-field, DAPI, mtGFP, and overlay (DAPI + mtGFP) confocal images. PU-91-treated AMD cybrids had a drastic increase in mtGFP fluorescence intensity compared to the untreated group (p≤0.05, n=3) (**B**). Data are presented as mean ±SEM and normalized to untreated (UN) AMD cybrids which were assigned a value of 1. Mann-Whitney test was used to measure statistical differences; *p≤0.05.

The cell biological features of mitochondria integrity are best observed in whole cells where mitochondria are visualized. Figure. 3A shows representative confocal images of AMD RPE cells stained with DAPI (blue) and mitochondrial GFP stain (green). Panel 1 shows bright-field images, panel 2 shows DAPI (blue)-stained images, panel 3 shows mtGFP (green)-stained images, and panel 4 shows merge (DAPI + mtGFP) images. The qualitative appearance of the mitochondrial network is vastly different in PU-91 treated cells compared with untreated controls (panel 3). When quantitated, PU-91-treated AMD cells showed an increase in mtGFP fluorescence intensity by 168% (p = 0.03; AMD UN: 1 ± 0.22, n=4; AMD PU-91: 2.68 ± 0.25, n=4) (Figure 3B) compared to the untreated AMD cells.

### PU-91 rescues AMD RPE cells from apoptotic cell death

**Figure 4:**
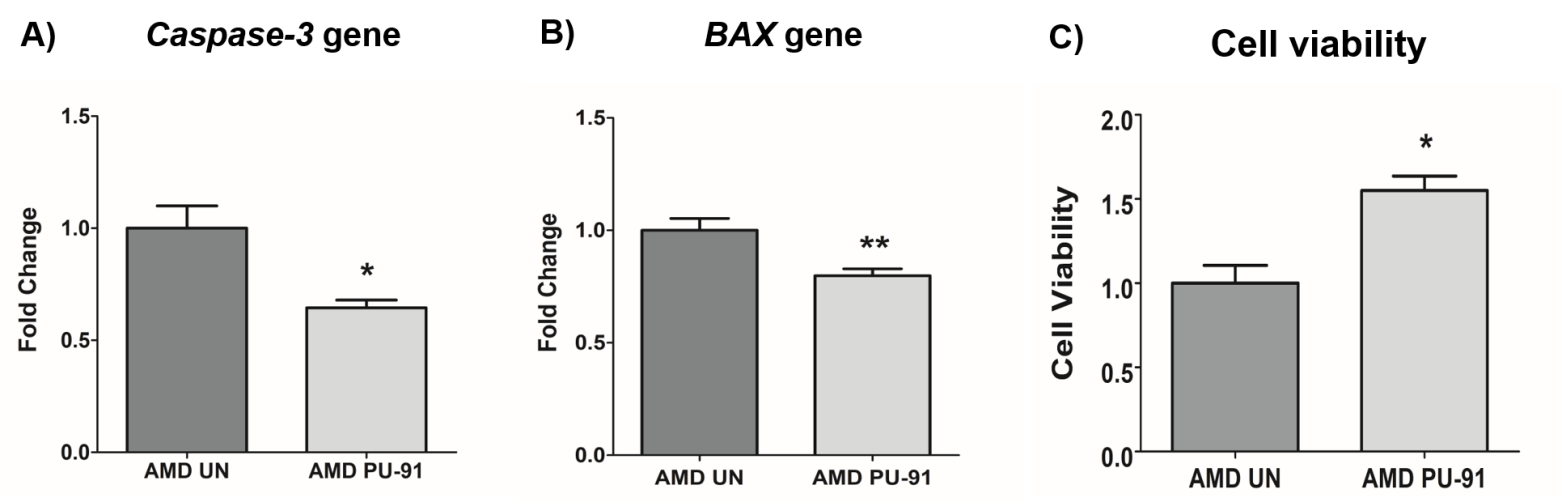
**PU-91 regulates apoptotic cell death.** qRT-PCR analysis showed downregulation of apoptotic genes such as *Caspase-3* (p≤0.05, n=4) (**A**) and *BAX* (p≤0.05, n=4) (**B**) in AMD cybrids treated with PU-91. Using the MTT assay, it was observed that PU-91-treated AMD cybrids had a higher number of viable cells compared to the untreated group (p≤0.05, n=4) (**C**). Data are presented as mean ± SEM and normalized to untreated (UN) AMD cybrids which were assigned a value of 1. Mann-Whitney test was used to measure statistical differences; *p≤0.05, **p≤0.01.

**Figure 5:**
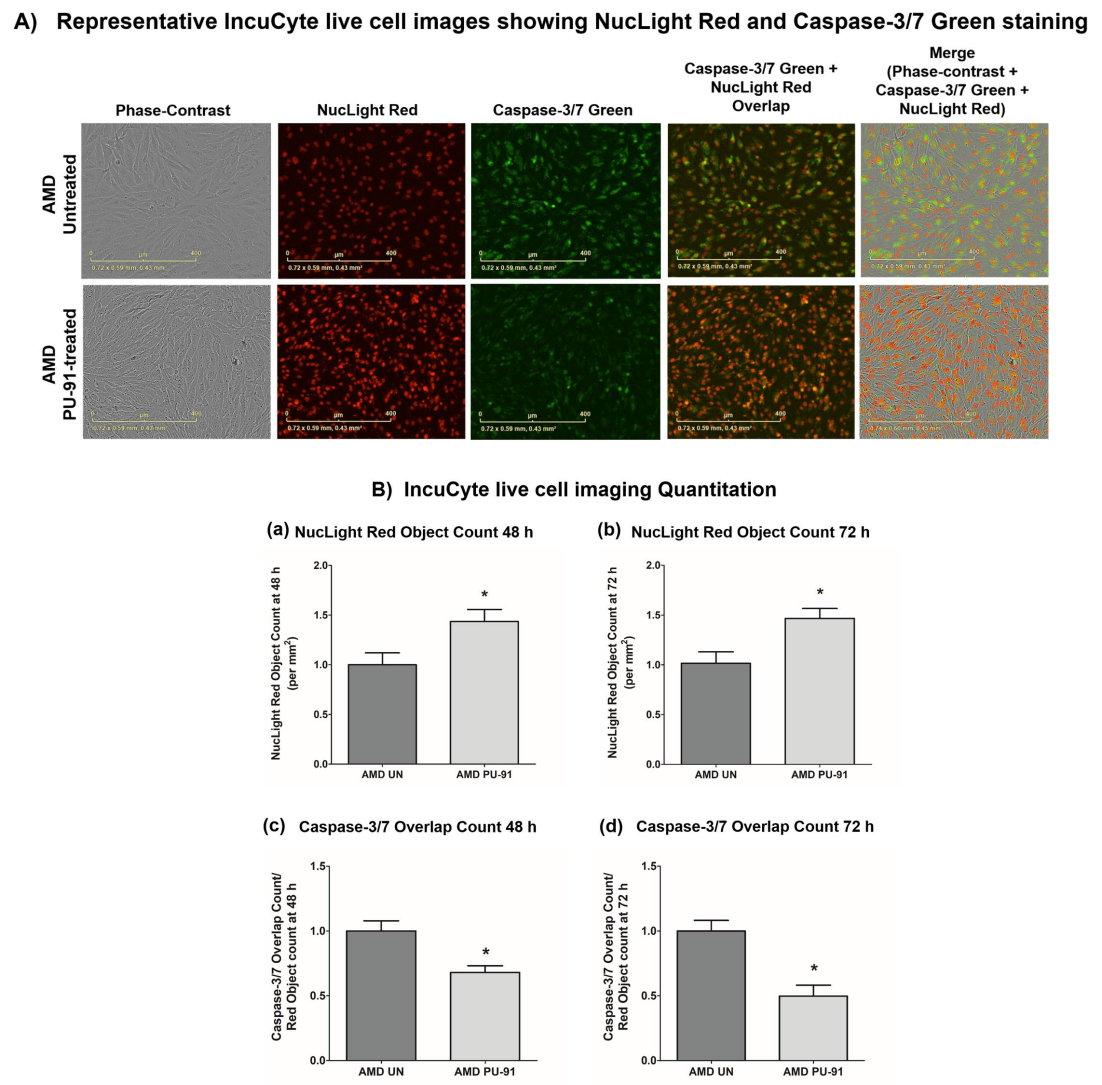
**PU-91 regulates apoptotic cell death – Caspase-3/7 staining.** (**A**) Shows representative IncuCyte live-cell images of untreated (AMD UN) and PU-91-treated AMD cybrid cells (AMD PU-91) stained with NucLight Red and Caspase-3/7 Green reagent. (**B**) Shows quantitation graphs for the 48 h and 72 h time points. Data are presented as mean ± SEM and normalized to untreated (UN) AMD cybrids which were assigned a value of 1. Mann-Whitney test was used to measure statistical differences; *p≤0.05.

Apart from promoting mitochondrial biogenesis, *PGC-1α* also partners to promote the expression of additional gene networks that are cytoprotective. Treatment of AMD RPE cells with PU-91 decreased expression of *Caspase-3* gene by 34% (p=0.016; AMD UN: 1 ± 0.099, n=5; AMD PU-91: 0.66 ± 0.03, n=5) (Figure 4A) and *BAX* gene by 21% (p=0.0079; AMD UN: 1 ± 0.05, n=5; AMD PU-91: 0.79 ± 0.03, n=5) (Figure 4B), and significantly increased cell viability by 55% (p=0.03; AMD UN: 1 ± 0.11, n=4; AMD PU-91: 1.55 ± 0.086, n=4) (Figure 4C) compared to their untreated counterparts. Furthermore, to examine and compare cell proliferation and apoptosis between untreated and PU-91-treated AMD cells, we performed IncuCyte® Live-Cell Imaging Analysis using Caspase- 3/7 Green and NucLight Red reagents (Figures 5A and 5B). Figure. 5A shows representative IncuCyte live-cell images. The upper panel represents untreated AMD group and the lower panel represents the PU-91-treated AMD group. Panel 1 shows phase-contrast images; panel 2 shows NucLight Red-stained images; panel 3 shows Caspase-3/7 Green-stained images; panel 4 shows Overlap i.e., Caspase-3/7 + NucLight Red images; and panel 5 shows Merged i.e., Phase-contrast + NucLight Red + Caspase-3/7 Green images. Compared to untreated AMD cells, PU-91-treated AMD cells showed 43.6% and 46.6% higher NucLight Red object count at the 48 h (p=0.03; AMD UN: 1 ± 0.119, n=4; AMD PU-91: 1.436 ± 0.119, n=4) (Figure 5B (a)) and 72 h (p=0.03; AMD UN: 1 ± 0.115, n=4; AMD PU-91: 1.466 ± 0.099, n=4) (Figure 5B (b)) timepoints respectively. Furthermore, as hypothesized, PU-91-treated AMD cells showed lower Overlap object count (i.e., Caspase-3/7 Green + NucLight Red staining (Yellow))/ Red object count compared to their untreated counterparts i.e., at 48 h *–* 32% decrease; p=0.03; AMD UN: 1 ± 0.079, n=4; AMD PU-91: 0.68 ± 0.05, n=4 (Figure 5B (c)) and at 72 h *–* 50.21% decrease; p=0.03; AMD UN: 1 ± 0.082, n=4, AMD PU-91: 0.498 ± 0.084, n=4 (Figure 5B (d)).

### PU-91 regulates inflammation and complement in AMD RPE cells

**Figure 6:**
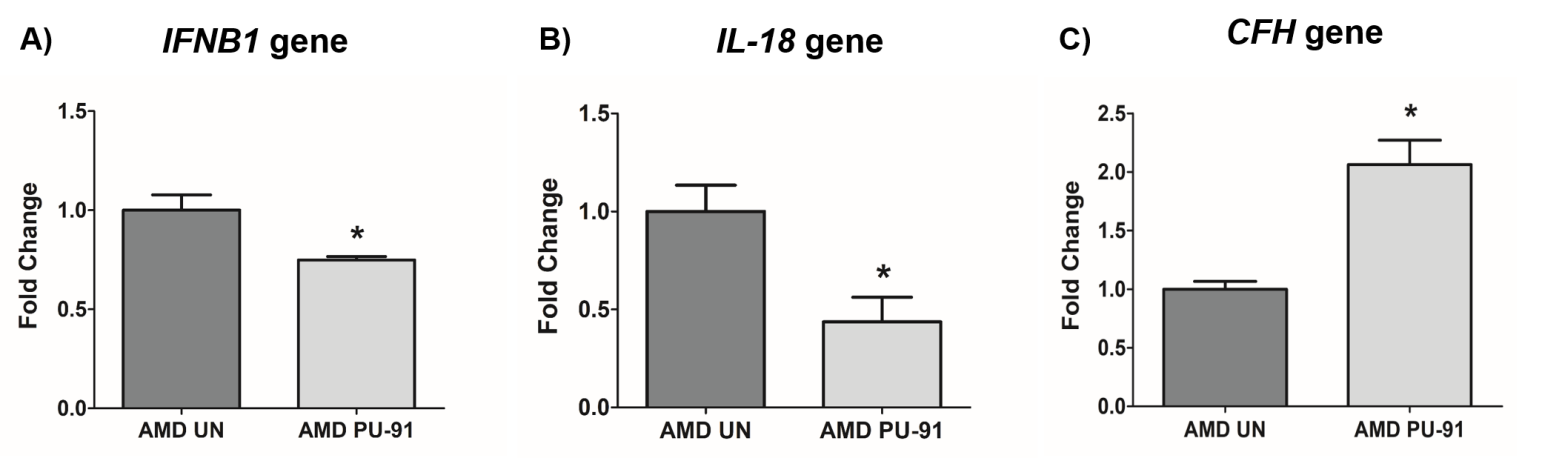
**PU-91 regulates inflammation and complement.** qRT-PCR analysis showed lower gene expression of inflammation markers such as *IFNB1* (p≤0.05, n=4) (**A**), *IL-18* (p≤0.05, n=4) (**B**) in PU-91-treated AMD cybrids (AMD PU-91) compared to untreated AMD cybrids (AMD UN). However, PU-91 upregulated the complement inhibitor *CFH* gene (p≤0.05, n=3-4) (**C**). Data are presented as mean ± SEM and normalized to untreated (UN) AMD cybrids which were assigned a value of 1. Mann-Whitney test was used to measure statistical differences; *p≤0.05.

Molecular correlates of AMD include chronic inflammation and dysregulation of the complement pathway. We sought to determine whether PU-91 regulated these pathogenic features. Treatment with PU-91 altered the gene expression of inflammatory markers, *IFNB1* (25% decrease; p=0.03; AMD UN: 1 ± 0.08, n=4; AMD PU-91: 0.75 ± 0.02, n=4) (Figure 6A), *IL-18* (56% decrease; p=0.03; AMD UN: 1 ± 0.13, n=4; AMD PU-91: 0.44 ± 0.12, n=4) (Figure 6B), and of a complement inhibitor *CFH* (106% increase; p=0.03; AMD UN: 1 ± 0.07, n=4; AMD PU-91: 2.06 ± 0.21, n=3) (Figure 6C).

### Additive effects of PU-91 + Esterase Inhibitors (EI) i.e., EI-12 and EI-78 on cell viability in AMD RPE cells

**Figure 7:**
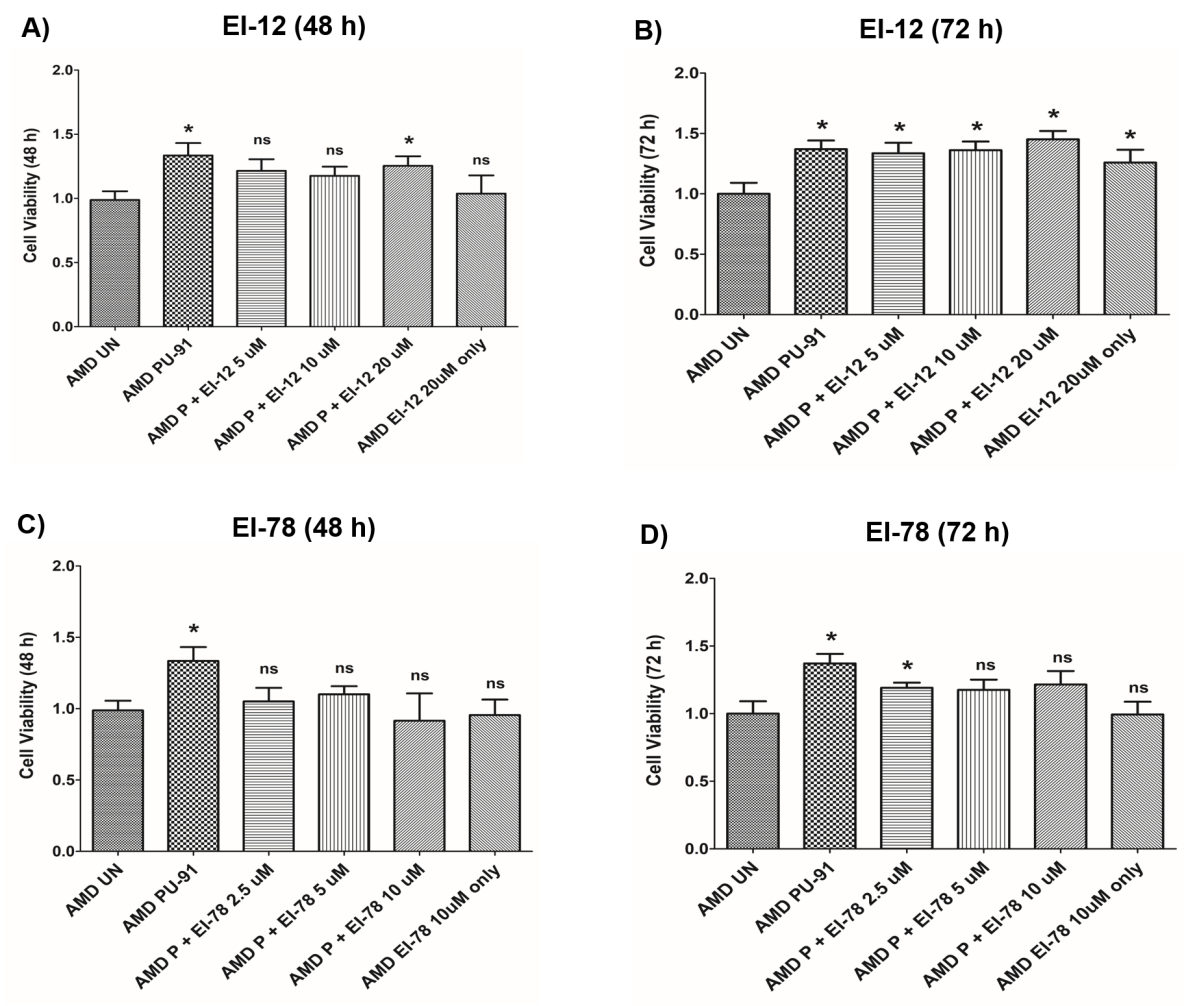
**Effect of PU-91 + EI-12/ EI-78 on cell viability.** This figure shows cell viability differences using MTT assay in AMD cells treated with P + EI-12 (**A** and **B**) / EI-78 (**C** and **D**) at 48 h and 72 h. Data (n=3) are presented as mean ± SEM and normalized to untreated (UN) AMD cybrids which were assigned a value of 1. Mann-Whitney test were used to measure statistical differences; *p≤0.05. P = PU-91; EI = Esterase Inhibitor.

To drive higher levels of PU-91 into tissues such as the neuroretina we evaluated co-administration with esterase inhibitors (EI) namely EI-12 and EI-78. To determine whether addition of either of these esterase inhibitors to PU-91 alters pharmacodynamic responses and/or produce independent effects, we studied these on AMD RPE cybrids. Figure 7A and 7B and Supplementary Table 2 show the effects of treatment with PU-91+EI-12 at varying concentrations i.e., EI-12 5 μΜ, 10 μΜ, and 20 μΜ, on the cell viability of AMD cybrid cells at 48 h (Figure 7A) and at 72 h (Figure 7B). Compared to untreated AMD cybrids, a significant increase in cell viability was observed in PU-91 treated AMD cybrids and in the PU-91+EI-12 20 μΜ group at 48 h.

Also, a significant difference in cell viability was observed between the untreated AMD group and the group treated with PU-91+EI-12 5 μΜ, PU-91+EI-12 10 μΜ and PU-91+ EI-12 20 μΜ at 72 h (Figure 7B). Compared to untreated AMD cells, treatment with PU-91+EI-78 at varying concentrations i.e., EI-78 2.5 μΜ, 5 μΜ and 10 μΜ, eliminated the cell viability cytoprotection of PU-91 alone on AMD RPE cells at 48 h (Figure 7C). Compared to untreated AMD cells, treatment with PU-91+EI-78 at concentrations 5 μΜ and 10 μΜ eliminated the cell viability cytoprotection of PU-91 alone on AMD RPE cells at 72 h time points. A significant increase in cell viability was observed between the untreated AMD group and the group treated with PU-91+EI-78 2.5 μΜ (Figure 7D, Supplementary Table 3).

No significant changes in cell viability were observed between AMD cells treated with only PU-91 and those treated with PU-91+EI-12 or PU-91+EI-78 (Supplementary Tables 2 and 3).

### Additive effects of PU-91 + EI-12 and EI-78 on gene expression in AMD RPE cells

We extended the observations of PU-91 with esterase inhibitors EI-12 and EI-78, on gene expression changes. Treatment with PU-91 + EI-12/ EI-78 at different concentrations i.e., EI-12: 5 μΜ, 10 μΜ, and 20 μΜ; EI-78: 2.5 μΜ, 5 μΜ, and 10 μΜ altered the expression of *PGC-1α, Caspase-3, IL-18, VEGF, SOD2* genes in AMD RPE cybrid cells (n=3) at the 72 h time point.

**Figure 8:**
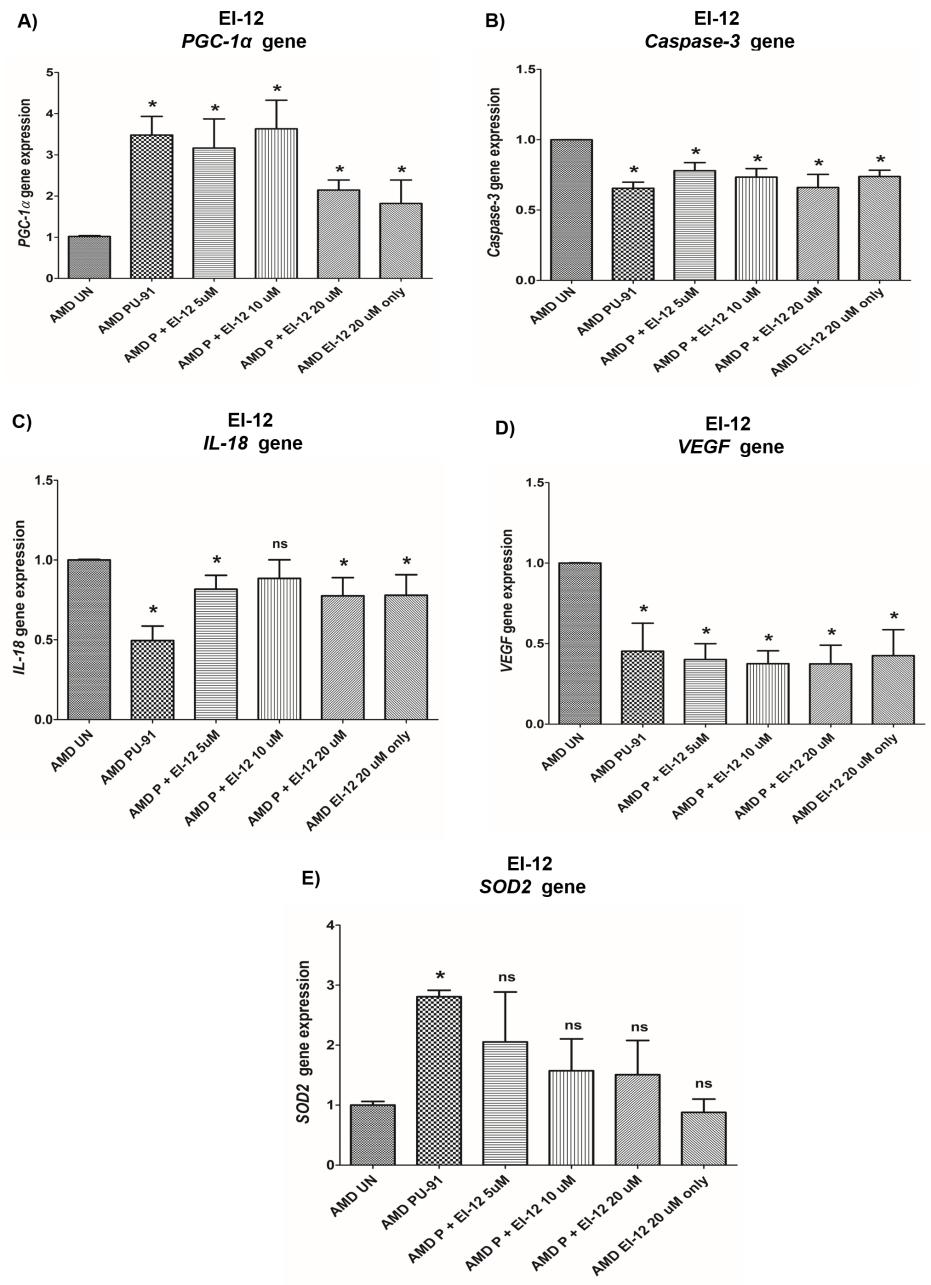
**Effect of PU-91 + EI-12 on gene expression.** qRT-PCR analysis showed differential expression of *PGC-1α* (**A**), *Caspase-3* (**B**), *IL-18* (**C**), *VEGF* (**D**), and *SOD2* (**E**) genes in AMD RPE cells at the 72 h time point. Data (n=3) are presented as mean ± SEM and normalized to untreated (UN) AMD cybrids which were assigned a value of 1. Mann-Whitney test was used to measure statistical differences; *p≤0.05. P = PU-91; EI = Esterase Inhibitor.

**Figure 9:**
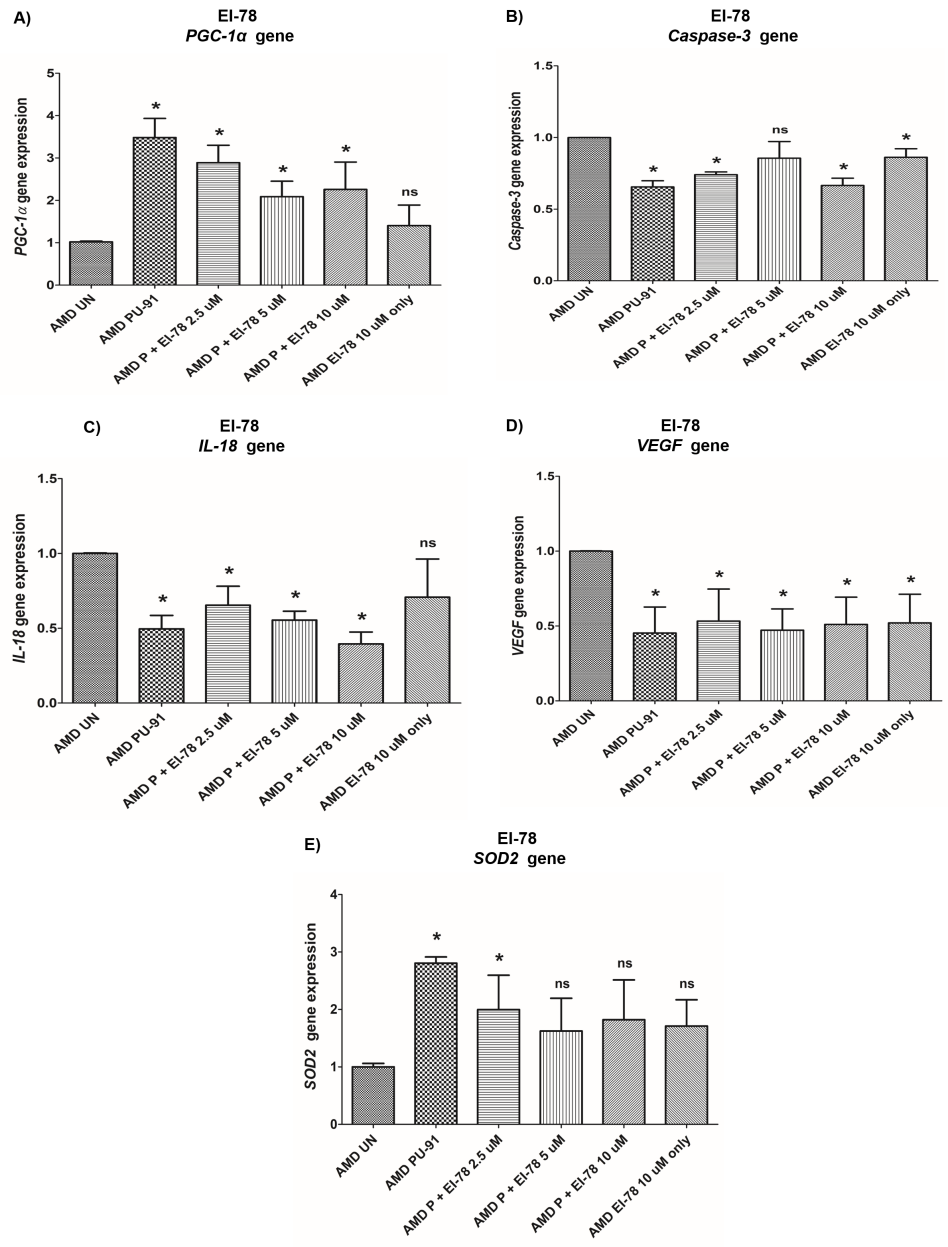
**Effect of PU-91 + EI-78 on gene expression.** qRT-PCR analysis showed differential expression of *PGC-1α* (**A**), *Caspase-3* (**B**), *IL-18* (**C**), *VEGF* (**D**), and *SOD2* (**E**) genes in AMD RPE cells at the 72 h time point. Data (n=3) are presented as mean ± SEM and normalized to untreated (UN) AMD cybrids which were assigned a value of 1. Mann-Whitney test was used to measure statistical differences; *p≤0.05. P = PU-91; EI = Esterase Inhibitor.

***PGC-1α*: EI-12 -** Compared to untreated AMD cybrids, significant *PGC-1α* upregulation was observed in PU-91-treated, P (PU-91)+EI-12 5 µM (216%), P+EI-12 10 µM (263%), P+EI-12 20 µM (115%) groups, and only EI-12 20 µM (82%) groups (Figure 8A, Supplementary Table 4). **EI-78 -** Compared to untreated AMD cybrids, significant *PGC-1α* upregulation was observed in PU-91-treated, P+EI-78 2.5 µM (189%), P+EI-78 5 µM (109%), and P+EI-78 10 µM (126%) groups, (Figure 9A, Supplementary Table 5).

***Caspase-3*: EI-12 -** Compared to untreated AMD cybrids, significant *Caspase-3* downregulation was observed in PU-91-treated, P+EI-12 5 µM (22%), P+EI-12 10 µM (27%), P+EI-12 20 µM (34%), and only EI-12 20 µM (26%) groups (Figure 8B, Supplementary Table 4). **EI-78 -** Compared to untreated AMD cybrids, significant *Caspase-3* downregulation was observed in PU-91-treated, P+EI-78 2.5 µM (26%), P+EI-78 10 µM (34%), and only EI-78 10 µM (13.8%) groups (Figure 9B, Supplementary Table 5).

***IL-18*: EI-12 -** Compared to untreated AMD cybrids, significant *IL-18* downregulation was observed only in the PU-91-treated group, P+EI-12 5 µM (18.3%), P+EI-12 20 µM (22.4%), and only EI-12 20 µM (22%) groups (Figure 8C, Supplementary Table 4). **EI-78 -** Compared to untreated AMD cybrids, significant *IL-18* downregulation was observed in PU-91-treated, P+EI-78 2.5 µM (34.6%), P+EI-78 5 µM (45%), and P+EI-78 10 µM (61%) groups (Figure 9C, Supplementary Table 5).

***VEGF*: EI-12 -** Compared to untreated AMD cybrids, significant *VEGF* downregulation was observed in PU-91-treated, P+EI-12 5 µM (60%), P+EI-12 10 µM (63%), P+EI-12 20 µM (63%), and only EI-12 20 µM (58%) groups (Figure 8D, Supplementary Table 4). **EI-78 -** Compared to untreated AMD cybrids, significant *VEGF* downregulation was observed in PU-91-treated, P+EI-78 2.5 µM (46.7%), and P+EI-78 5 µM (53%), P+EI-78 10 µM (48.9%), and only EI-78 10 µM (47.9%) groups (Figure 9D, Supplementary Table 5).

***SOD2*: EI-12 -** Compared to untreated AMD cybrids, significant *SOD2* upregulation was observed only in the PU-91-treated group (Figure 8E, Supplementary Table 4). **EI-78 -** Compared to untreated AMD cybrids, significant *SOD2* upregulation was observed only in the PU-91-treated and P+EI-78 2.5 µM (99.7%) groups (Figure 9E, Supplementary Table 5).

## DISCUSSION

We report that PU-91, an FDA-approved drug, promotes mitochondrial-stabilization, *PGC-1α* upregulation, cytoprotection of AMD ARPE-19 transmitochondrial cybrid cells by preserving mitochondrial health, reducing apoptotic cell loss, and inducing upregulation of the MDP-coding *MT-RNR2* gene. Our study establishes PU-91 as a potential candidate drug for AMD therapy.

Gene expression analyses revealed significant upregulation of mitochondrial biogenesis pathway genes i.e., *PGC-1α, NRF-1, NRF-2, PPAR-α,* and *PPAR-γ* in PU-91-treated AMD cybrids. PU-91 positively regulates the expression of nucleus-encoded markers of mitochondrial biogenesis in AMD mitochondria-containing cybrid cell lines. Dysregulation of mitochondrial function and decreased PGC-1α levels have been implicated in neurodegeneration [16,17]. PGC-1α is a master regulator of mitochondrial biogenesis in several disease models including cardiac disorders, Parkinson’s disease, Huntington’s disease and Alzheimer’s disease [18–24]. A study demonstrated that PGC-1α activity and signaling are induced to regulate detoxifying responses to oxidative and metabolic stress in inner retina [25]. The same study identified PGC-1α as an important regulator of retinal ganglion cell (RGC) homeostasis and astrocyte reactivity. Moreover, PGC-1α, by regulating senescence, protects RPE cells against oxidative damage in aging retina in AMD-like pathology *in vivo* [26]. Iacovelli et al. reported major involvement of PGC-1α in mitochondrial function and in antioxidant capacity in primary human RPE cells and in ARPE-19 cell lines *in vitro* [13]. PGC-1α increases the expression of mitochondrial enzymes such as ATP synthase and Cytochrome c Oxidase (COX), and induces mitochondrial biogenesis by activating various transcription factors, including NRF-1 and NRF-2, which in turn induce TFAM, PPARs, estrogen and ERRs [27].

NRF-1 and NRF-2 are redox-sensitive transcription factors that are activated in response to oxidative stress. By inducing cytoprotective molecules, they orchestrate a defense mechanism against reactive oxygen species (ROS)-induced cytotoxicity. Moreover, NRF-1 and NRF-2 are known to protect neurons against acute brain injury [28]. NRF2 binds to the antioxidant response element (ARE) in the promoter regions of its target genes, thereby activating antioxidant gene transcription [29]. Activation of NRF-2 conferred neuroprotective effects in the retina post-ischemia/reperfusion injury *in vivo* [30]. Both NRF-1 and NRF-2 activate the genes involved in oxidative homeostasis. Deletion of *PGC-1α* and *NRF-2,* as found in NRF-2/PGC-1α dKO mice, resulted in significant age-dependent RPE degeneration [31]. Substantial *in vivo* evidence demonstrates the involvement of NRF-1 and NRF-2 in early development, and their absence causes embryonic lethality and oxidative stress-induced toxicity [32]. NRF-1 activates the *Cytochrome c* gene and therefore plays a role in nuclear-mitochondrial interactions [33].

PPARs, a subfamily of nuclear receptors, are transcription factors that can heterodimerize with partners, such as the retinoid X receptor (RXR) and bind to DNA of target genes [34]. Ding et al. demonstrated that PPAR-α (Peroxisome Proliferator-Activated Receptor α) improves mitochondrial oxygen consumption and protects capillary pericytes in the retina [35] Pearsall et al. showed that PPAR-α plays a pivotal role in retinal neuronal survival, lipid metabolism and improves retinal energy efficiency. Since energy deficits due to dysfunctional mitochondria have been implicated in AMD, PPAR-α was suggested to be a putative therapeutic target in AMD [36]. Zhu et al. reported a potential role of PPAR-γ in differentiation and maturation of retinal cells [37]. Studies also highlighted potential neuroprotective function of PPAR-γ agonists *in vivo* and *in vitro* in neurodegenerative diseases such as Alzheimer’s [38], Parkinson’s [39], and ALS [40]. Addition of the PPAR-γ agonists such as Pioglitazone is known to induce neuronal survival and protection from apoptotic cell death in the retina [41]. Furthermore, PPAR-γ ligands inhibit corneal neovascularization [42–44] and injury-induced scarring in the conjunctiva after glaucoma surgery [45]. Murata et al. demonstrated inhibition of choroidal angiogenesis and VEGF-induced RPE migration and proliferation *in vitro* by PPAR-𝛾 ligands namely troglitazone/ rosiglitazone. This group also showed that intravitreal injection of troglitazone caused significant reduction in lesions and leakage in the eyes of choroidal neovascular (CNV) animal models [46]. In summary, by modulating the expression of mitochondrial biogenesis mediators, PU-91 orchestrates mitochondrial and cellular health.

We next performed JC-1 dye assay to compare mitochondrial membrane potential (ΔΨm) between untreated and PU-91-treated AMD cybrids. Previously, it has been established that any imbalance in oxidative redox state causes mitochondrial depolarization, opening of mitochondrial permeability transition pore, and collapse of mitochondrial membrane potential, subsequently leading to apoptosis [47,48]. These events hold true in the retina as well [49]. We know that AMD cybrids have damaged mitochondria, which likely contribute to compromised mitochondrial membrane potential. However, PU-91 administration improved ΔΨm significantly in AMD cybrid cells, suggesting that PU-91 can protect mitochondrial membrane integrity and function. Potential mechanisms of PU-91-mediated ΔΨm restoration including attenuation of mitochondrial depolarization should be further examined. Consistent with our study, Chong et al. demonstrated that Artemisinin, an FDA-approved drug improves ΔΨm as measured by JC-1 assay and protects human retinal pigment epithelial cells from oxidative damage [50]. Moreover, *in vivo* studies by Ellis et al. showed that addition of Sigma-1 receptor agonists restored ΔΨm in oxygen-deprived retina [51].

Mitochondria are a major source of reactive oxygen species (ROS) in a cell, and the principal mitochondrial ROS is superoxide anion, which is a by-product of leakage from the Electron Transport Chain. We have previously shown that AMD RPE cybrid cells have higher mitochondrial superoxide production compared to age-matched normal cybrid cells [9]. In the current study, we used a fluorogenic MitoSOX Red dye for specific detection of mitochondrial superoxide. Once inside the mitochondria, the MitoSOX Red reagent is oxidized by superoxide and exhibits red fluorescence. Our results revealed significantly diminished mitochondrial superoxide production in PU-91-treated AMD cybrids compared to untreated AMD cybrids. This finding is important because elevated levels of intracellular ROS and mitochondrial superoxides contribute to retinal and neurological diseases [52–54], and reduction in mitochondrial superoxide is critical to protect against oxidative stress-related diseases.

We also examined in AMD cybrids the gene expression of *SOD2,* the mitochondrial Super Oxide Dismutase, also known as MnSOD (Manganese SOD). SOD2 prevents binding of mitochondrial superoxide to nitric oxide thereby preventing apoptosis, necrosis, mitophagy, and autophagy [55]. Deficiency of SOD2 causes extensive oxidative damage in the RPE and has been associated with AMD pathogenesis [56]. Recent Genome-Wide Association Studies (GWAS) have suggested an association between a susceptible locus - rs2842992 near the SOD2 gene and geographic atrophy in AMD [57]. In the present study, PU-91 treatment upregulated *SOD2* gene levels significantly, which would enhance the antioxidant effects in AMD cybrids. Hypoxic stress and activation of HIF1α has been implicated in AMD. ROS and HIF1α cause VEGF activation thereby triggering angiogenesis and subsequent choroidal neovascularization in wet AMD [58,59]. PU-91-treated AMD cybrids had lower expression of *HIF1α* gene, suggesting that PU-91 may exhibit hypoxia-suppressing effects. Cumulatively, these results highlight a key pharmacological role of PU-91 in decreasing oxidative stress in AMD cells.

We have established previously that dysfunctional mitochondria in the AMD cybrids contribute to activation of cleaved Caspase-3 and BAX, which are markers of cell apoptosis [9]. Other studies have demonstrated the role of apoptotic and necrotic cascades in death of retinal cells in AMD [60,61]. Therefore, treatment with drugs that inhibit apoptosis is essential to prevent retinal cell loss and to preserve cellular heath. We observed higher number of viable cells and downregulation of *Caspase-3* and *BAX* genes in PU-91-treated AMD cybrids, indicating that PU-91 prevents mitochondria-induced apoptotic cell death in these cells. Next, we compared cell proliferation and apoptosis between untreated and PU-91-treated AMD cells using IncuCyte® Live-Cell Imaging Analysis system and Caspase- 3/7 Green and NucLight Red reagents. PU-91-treated AMD cells showed significantly higher NucLight Red object count at the 48 h and 72 h compared to untreated AMD cells. Furthermore, as hypothesized, PU-91-treated AMD cells showed lower Overlap object count (i.e., Caspase-3/7 Green + NucLight Red staining (Yellow))/ Red object count compared to their untreated counterparts. Our IncuCyte data revealed that PU-91 enhanced cell proliferation and reduced Caspase-3/7 activity in AMD cybrids. To our knowledge, this is the first study to report these findings.

The *MT-RNR2* gene contains small open-reading frames for mitochondrial-derived peptides (MDPs) such as Humanin and SHLPs that possess cytoprotective and neuroprotective properties [9,62,63]. Interestingly, addition of PU-91 upregulated the MDP-coding *MT-RNR2* gene in AMD cybrids, suggesting that PU-91-mediated protective effects in AMD cybrids may be partly attributed to MDP production. Therefore, PU-91 may ameliorate cell health by triggering production of MDPs including Humanin, which is cytoprotective in AMD [9].

Mitochondrial stabilization and protection are potential mechanisms by which PU-91 protects AMD RPE cybrids. To compare mitochondrial density between untreated and PU-91-treated AMD cells, we transduced the cells with CellLight reagent, which is a GFP-E1 alpha pyruvate dehydrogenase leader peptide construct driven by a mammalian promoter. This fluorescent fusion construct provides precise targeting to mitochondria. Herein, treatment with PU-91 enhanced mitochondrial GFP fluorescence appreciably in AMD cells compared to their untreated counterparts, indicating that PU-91 can augment and/or prevent mitochondrial loss in AMD cybrids. These results are consistent with a previous study wherein Humanin G, a mitochondrial-derived peptide, rescued AMD mitochondria in RPE cybrid cells [9].

PU-91 attenuated *IL-18* gene expression, thereby reducing mtDNA damage-induced inflammation in AMD cybrids. This is significant because elevation of pro-inflammatory cytokines in the serum and ocular fluids of AMD patients accompanies pathogenesis. Ijima et al. suggested association of IL-18 with dry AMD since patients with dry AMD had higher IL-18 serum levels; this study also demonstrated IL-18-induced RPE cell degeneration in mouse eye [64]. AMD cybrids treated with PU-91 showed reduced expression of *IFNB1* gene which has been demonstrated to reduce human RPE cell proliferation [65]. As shown previously, AMD cybrids have decreased expression of CFH, an inhibitor of complement pathway, consistent with complement activation [66]. Moreover, AMD patients carrying the high-risk allele for CFH showed substantial retinal mtDNA damage [67]. MtDNA dysfunction has been associated with AMD due to increased mtDNA lesions with aging [68]. Significant increase in *CFH* gene expression was observed in PU-91-treated AMD cybrids, suggesting inhibition of complement by PU-91.

Next, we investigated the effects of co-administration of PU-91 with esterase inhibitors (EI) - EI-12 and EI-78, which are being evaluated to augment PU-91 penetration into retina and neural tissue *in vivo*. Administration of PU-91 in humans/animals results in a large first pass effect, converting the vast majority of PU-91 to its primary metabolite, PU-91*, which is *inactive* as a PGC-1α upregulator. We identified the mechanism of PU-91 to PU-91* conversion and identified two esterase inhibitors namely EI-12 and EI-78, that when co-administered with PU-91 largely block conversion to PU-91*, thereby markedly increasing CNS bioavailability of PU-91. We tested the following co-administration combinations – 1) PU-91 50 μM + EI-12 5 μM, 2) PU-91 50 μM + EI-12 10 μM, 3) PU-91 50 μM + EI-12 20 μM, 4) PU-91 50 μM + EI-78 2.5 μM, 5) PU-91 50 μM + EI-78 5 μM, 6) PU-91 50 μM + EI-78 10 μM, 7) EI-12 20 μM, 8) EI-78 10 μM, 9) PU-91 50 μM, and 10) AMD untreated. No substantive changes either in cell viability or gene expression (*PGC-1α*, *Caspase-3*, *IL-18*, *VEGF*, and *SOD2*) were observed when treated with a combination of PU-91 + EI-12/EI-78 compared to treatment with PU-91 alone.

In summary, in the *in vitro* AMD RPE transmitochondrial cybrids, the PU-91 drug: 1) regulates the mitochondrial biogenesis pathway, 2) improves mitochondrial function, 3) enhances mitochondrial GFP fluorescence, 4) prevents apoptotic cell death, 5) favorably regulates inflammation and complement, 6) favorably regulates the MDP-coding *MT-RNR2* gene, 7) when co-administered with EI-12/EI-78, does not impact either the viable cell count or gene expression (*PGC-1α*, *Caspase-3*, *IL-18*, *VEGF*, and *SOD2*) substantially compared to treatment with PU-91 alone.

In conclusion, PU-91 rescues AMD RPE cybrids, and potentially could be repurposed as an FDA-approved drug to prevent/treat AMD. Since it improves mitochondrial function and has already been FDA-approved, the candidate therapeutic PU-91 will be an excellent treatmentoption for AMD. Repositioning of PU-91 will be a smoother transition from lab bench to clinic since the pharmacological profiles of PU-91 have been examined already. Furthermore, because of its extensive safety record it could be potentially prosecuted through NDA more rapidly than a drug-like new chemical entity. Bringing a disease modifying therapeutic to market for the most prevalent form of blindness, AMD, has substantial potential benefit for our aging populations world-wide.

## MATERIALS AND METHODS

### Human subjects

The University of California Irvine’s Institutional review board approved research with human subjects (Approval #2003–3131). All participants provided informed consent and clinical investigations were performed according to the tenets of Declaration of Helsinki.

### Cell culture

Passage 5 AMD ARPE-19 transmitochondrial cybrids were created as described previously [9]. Briefly, these cybrid cells were prepared by polyethylene glycol fusion of mitochondrial DNA-deficient ARPE-19 (*Rho^0^*) cell line with platelets isolated from AMD patients. All cybrids used in this study (Supplementary Table 1) belonged to the ‘H’ mtDNA haplogroup. Our ARPE-19 cells have been validated using RPE-specific markers such as Bestrophin 1, Cellular retinaldehyde binding protein-1, and Keratin-18. Cybrid status and that the cybrids have acquired their mtDNAs from the donor individuals was confirmed using allelic discrimination, Sanger Sequencing, and Next-Generation Sequencing.

***Culture conditions***: The base medium for this cybrid cell line is DMEM-F12 Medium (Cat. # 10-092CM, Fisher Scientific, Pittsburgh, PA). DMEM-F12 Medium contains 3.15 g/L D-glucose, 2.5 mM L-glutamine, 15 mM HEPES, 0.5 mM sodium pyruvate, and 1200 mg/L sodium bicarbonate. To make the complete growth medium, fetal bovine serum was added to the base medium to a final concentration of 10%.

### Treatment with PU-91 and esterase inhibitors (EI-12 and EI-78)

PU-91 stock solution of 40 mM concentration was prepared at 15 mg/mL in DMSO. PU-91 stock was diluted in culture media to obtain a working concentration of 50 µM, which was used for all experiments in this study. PU-91 has been estimated to have clinical exposure in greater than 5 million patient years. 50 μM was selected as this concentration produces an optimal response in cell culture studies. Stock solutions of 20 mM EI-12 and 10 mM EI-78 were prepared in DMSO and were diluted in culture media to obtain the following working concentrations: EI-12 at 5 μM, 10 μM, and 20 μM; EI-78 at 2.5 μM, 5 μM, and 10 μM.

### Mitochondrial copy number

Total DNA was extracted from AMD cybrids followed by quantitative real-time PCR (qRT-PCR). QRT-PCR was performed using TaqMan gene expression assays for *18S* and *MT-ND2* (Cat. # 4331182, Thermo Fisher Scientific) genes and TaqMan gene expression master mix (Cat. # 4369016, Thermo Fisher Scientific). Relative mtDNA copy numbers were determined using delta Cts.

### Quantitative Real-Time PCR

RNA extraction, cDNA synthesis, and qRT-PCR analysis were performed as described previously [9]. QuantiTect Primer Assays were used to study the expression of *Caspase-3* gene (Cat. # QT00023947, Qiagen, Germantown, MD), *BAX* gene (Cat. # QT00031192, Qiagen), *HIF1α* gene (Cat. # QT00083664, Qiagen), *CFH* gene (Cat. # QT00001624, Qiagen), and *SOD2* gene (Cat. # QT01008693, Qiagen). KiCqStart® SYBR® green primers were used to examine the expression of *PGC-1α*, *NRF-1, NRF-2, PPAR-α, PPAR-γ, VEGF, IL-18,* and *IFNB1* genes (Cat. # kspq12012, Sigma, St. Louis, MO). Specific housekeeper genes used were *HPRT1* (Cat. # QT00059066, Qiagen) for *Caspase-3, BAX, SOD2, VEGF, IL-18, NRF-1, NRF-2, PPAR-α,* and *PPAR-γ*; *HMBS* (Cat. # QT00014462) for *CFH, PGC-1α* and TUBB (Cat. # QT00089775, Qiagen) for *HIF-1α*. TaqMan gene expression master mix (Cat. # 4369016, Life Technologies) and TaqMan gene expression assays were used to examine the expression of the *MT-RNR2* gene (Assay ID: Hs02596860_s1, Life Technologies), for which *GAPDH* (Assay ID: Hs02786624_g1, Life Technologies) was used as a housekeeper gene. Data analysis was performed using ∆∆Ct method. ∆Ct was the difference between the Cts (threshold cycles) of the target gene and Cts of the housekeeper gene (reference gene). Fold change was calculated using the following formula: Fold change = 2^ΔΔCt^.

### Cell viability assay

The numbers of viable cells were measured using the MTT (3-(4,5-dimethylthiazol-2-yl)-2,5-diphenyltetrazolium bromide) assay. Cells were plated in 96-well tissue culture plates, treated with 50 µM PU-91 followed by addition of MTT. Cells were incubated at 37 °C for 1 h, followed by addition of DMSO. Absorbance was measured at 570 nm and background absorbance measured at 630 nm. Normalized absorbance values were obtained by subtracting background absorbance from signal absorbance. The colorimetric signal obtained was proportional to the cell number.

### IncuCyte live-cell imaging

IncuCyte live-cell imaging was performed as described previously [69]. The IncuCyte NucLight Rapid Red Reagent is a cell permeable DNA stain that specifically stains nuclei in live cells and enables real-time quantification of cell proliferation. Addition of this reagent to normal healthy cells does not interfere with cell growth and morphology and provides homogenous staining of nuclei. In the culture medium, this inert stain crosses the cell membrane and has excellent specificity for DNA without the need for a wash step.

The IncuCyte Caspase-3/7 Green Apoptosis Reagent couples the activated Caspase-3/7 recognition motif (DEVD) to a DNA intercalating dye and enables real-time quantification of cells undergoing caspase-3/7 mediated apoptosis. This reagent is an inert, non-fluorescent substrate which when added to culture medium, crosses the cell membrane where it is cleaved by activated caspase-3/7 resulting in the release of the DNA dye and fluorescent staining of the nuclear DNA.

Cells were seeded in 96-well plates at a density of 5,000 – 10,000 cells/well followed by staining with IncuCyte® NucLight Rapid Red (1:500) and Caspase-3/7 Green (1:1000) labeling reagents. Stained cell plates were placed into the IncuCyte® live-cell analysis system and allowed to warm to 37 °C for 30 min prior to scanning. Phase Contrast, Green, and Red channels were selected, 5 images were taken per well with an average scan interval of 2 h until the experiment was complete. Fluorescent objects were quantified using the IncuCyte® integrated analysis software that minimizes background fluorescence.

### Mitochondrial membrane potential assay

The JC-1 assay uses a unique cationic dye i.e., 5,5’,6,6’-tetrachloro-1,1’,3,3’- tetraethylbenzimidazolylcarbocyanine iodide, to detect loss of mitochondrial membrane potential. JC-1 1X reagent was prepared by diluting 100X JC-1 reagent in assay buffer to 1:100 dilutions. AMD cybrids were plated in 24-well tissue culture plates for 24 h followed by treatment with 50 µM PU-91. 1X JC-1 reagent was added to cells and incubated for 15 min at 37 °C. JC-1 reagent in the wells was then replaced with DPBS and fluorescence was measured as follows: Red fluorescence (Live cells): Excitation 550 nm and Emission 600nm; Green fluorescence (Apoptotic cells): Excitation 485 nm and Emission 535 nm. Ratio of Red/Green was used for analysis. Lower ratio corresponded to higher apoptotic/dead cell number.

### MitoSOX assay

The fluorogenic MitoSOX Red dye (Cat. # M36008, Invitrogen, Grand Island, NY, USA) is a live-cell permeant reagent that detects mitochondrial superoxide in cells. MitoSOX Red reagent oxidized by superoxide has red fluorescence that can be quantified. AMD cybrids were plated in 24-well tissue culture plates. Stock solution of 5 mM MitoSOX reagent was diluted with HBSS (Hank’s balanced salt solution) buffer to obtain a 5 μM working solution. Cells were treated with 5 μM MitoSOX reagent and incubated for 10 min at 37 °C. Cells were then washed with HBSS buffer, and fluorescence was measured at excitation/emission maxima of 510/580 nm.

### CellLight mitochondrial GFP staining and confocal microscopy

Staining with CellLight Mitochondrial GFP probe (Cat. # C10600, Thermo Fisher Scientific, MA, USA) and confocal microscopy were performed as described previously [9]. Cells were plated in 4-well tissue culture chamber slides, stained with CellLight mtGFP for 24 h and incubated overnight at 37 °C. The cells were washed with 1X TBS (Tris buffered saline), fixed in paraformaldehyde and mounted in DAPI. Confocal z-stack images were captured using the LSM-700 Confocal microscope (Zeiss, Thornwood, NY, USA). ZEN 2 lite software (Zeiss) was used for fluorescence quantitation.

### Statistical analysis

Non-parametric Mann-Whitney test (GraphPad Prism 5.0; GraphPad Software, CA, USA) was used to analyze data between groups and to determine significance. p ≤ 0.05 was statistically significant.

## SUPPLEMENTARY MATERIALS

Supplementary Tables
