# Supplementary material for: PU-91 drug rescues human age-related macular degeneration RPE cells; implications for AMD therapeutics
Source: Aging (Albany NY). 2019 Sep 2;11(17):6691–713. doi: 10.18632/aging.102179 (PMC6756897; doi:10.18632/aging.102179)
Supplement: Supplementary Tables [file aging-11-102179-s001.pdf]

SUPPLEMENTARY TABLES

Supplementary Table 1. AMD Patient/ Cybrid cell lines' Patient Information.

CYBRID NUMBER	CYBRID TYPE	AGE	GENDER	RACE	HAPLOGROUP
1	AMD	81	Female	White	H
2	AMD	82	Female	White	H
3	AMD	86	Female	White	H
4	AMD	84	Female	White	H
5	AMD	75	Female	White	H
6	AMD	76	Male	White	H
7	AMD	83	Male	White	H
8	AMD	74	Male	White	H
9	AMD	76	Male	White	H
10	AMD	83	Male	White	H

This table provides information about the AMD patients and AMD cybrid cell lines.

Supplementary Table 2. Effects of PU-91 + EI-12 on cell viability.

Effect of EI-12 on cell viability 48 hr Time point	AMD UN	AMD Only PU-91-treated	AMD PU-91 + EI-12 5 µM	AMD PU-91 + EI-12 10 µM	AMD PU-91 + EI-12 20 µM	AMD Only EI-12 20 µM
Mean ± SEM	1 ± 0.07	1.33 ± 0.09	1.215 ± 0.09027	1.176 ± 0.07125	1.253 ± 0.07453	1.037 ± 0.1413
Effect of EI-12 on cell viability 72 hr Time point	AMD UN	AMD Only PU-91-treated	AMD PU-91 + EI-12 5 µM	AMD PU-91 + EI-12 10 µM	AMD PU-91 + EI-12 20 µM	AMD Only EI-12 20 µM
Mean ± SEM	1 ± 0.09	1.37 ± 0.07	1.335 ± 0.08792	1.361 ± 0.07264	1.452 ± 0.06857	1.259 ± 0.1060

This table shows the additive effects of PU-91 + EI-12 on cell viability. All values are normalized to 1 and are presented as Mean ± SEM.

Supplementary Table 3. Effects of PU-91 + EI-78 on cell viability.

Effect of EI-78 on cell viability 48 hr Time point	AMD UN	AMD Only PU-91-treated	AMD PU-91 + EI-78 2.5 µM	AMD PU-91 + EI-78 5 µM	AMD PU-91 + EI-78 10 µM	AMD Only EI-78 10 µM
Mean ± SEM	1 ± 0.07	1.33 ± 0.09	1.05 ± 0.09	1.10 ± 0.06	0.91 ± 0.19	0.95 ± 0.11
**Effect of EI-78 on cell viability 72 hr Time point**	**AMD UN**	**AMD Only PU-91-treated**	**AMD PU-91 + EI-78 2.5 µM**	**AMD PU-91 + EI-78 5 µM**	**AMD PU-91 + EI-78 10 µM**	**AMD Only EI-78 10 µM**
Mean ± SEM	1 ± 0.09	1.37 ± 0.07	1.19 ± 0.04	1.17 ± 0.08	1.21 ± 0.09	0.99 ± 0.09

This table shows the additive effects of PU-91 + EI-78 on cell viability. All values are normalized to 1 and are presented as Mean ± SEM.

Supplementary Table 4. Effects of PU-91 + EI-12 on gene expression.

Effect of EI-12 on gene expression		AMD UN	AMD Only PU-91-treated	AMD PU-91 + EI-12 5 µM	AMD PU-91 + EI-12 10 µM	AMD PU-91 + EI-12 20 µM	AMD Only EI-12 20 µM
PGC-1α	Mean ± SEM	1 ± 0.02	3.48 ± 0.45	3.16 ± 0.71	3.63 ± 0.69	2.15 ± 0.24	1.82 ± 0.57
Caspase-3	Mean ± SEM	1 ± 0.0002	0.65 ± 0.04	0.78 ± 0.06	0.73 ± 0.06	0.66 ± 0.09	0.74 ± 0.045
IL-18	Mean ± SEM	1 ± 0.004	0.49 ± 0.09	0.82 ± 0.09	0.88 ± 0.12	0.77 ± 0.11	0.78 ± 0.13
VEGF	Mean ± SEM	1 ± 0.002	0.45 ± 0.17	0.40 ± 0.09	0.37 ± 0.08	0.37 ± 0.12	0.42 ± 0.16
SOD2	Mean ± SEM	1 ± 0.06	2.81 ± 0.11	2.05 ± 0.83	1.57 ± 0.53	1.51 ± 0.57	0.88 ± 0.22

This table shows the additive effects of PU-91 + EI-12 on gene expression. All values are normalized to 1 and are presented as Mean ± SEM.

Supplementary Table 5. Effects of PU-91 + EI-78 on gene expression.

Effect of EI-78 on gene expression		AMD UN	AMD Only PU-91-treated	AMD PU-91 + EI-78 2.5 μM	AMD PU-91 + EI-78 5 μM	AMD PU-91 + EI-78 10 μM	AMD Only EI-78 10 μM
PGC-1α	Mean ± SEM	1 ± 0.02	3.48 ± 0.45	2.89 ± 0.41	2.09 ± 0.37	2.26 ± 0.64	1.40 ± 0.48
Caspase-3	Mean ± SEM	1 ± 0.0002	0.65 ± 0.04	0.74 ± 0.019	0.85 ± 0.12	0.66 ± 0.05	0.86 ± 0.06
IL-18	Mean ± SEM	1 ± 0.004	0.49 ± 0.09	0.65 ± 0.13	0.55 ± 0.06	0.39 ± 0.08	0.71 ± 0.25
VEGF	Mean ± SEM	1 ± 0.002	0.45 ± 0.17	0.53 ± 0.21	0.47 ± 0.14	0.51 ± 0.18	0.52 ± 0.19
SOD2	Mean ± SEM	1 ± 0.06	2.81 ± 0.11	1.99 ± 0.59	1.62 ± 0.57	1.82 ± 0.69	1.71 ± 0.46

This table shows the additive effects of PU-91 + EI-78 on gene expression. All values are normalized to 1 and are presented as Mean ± SEM.
